# Effect of lesser trochanter posteromedial wall defect on the stability of femoral intertrochanteric fracture using 3D simulation

**DOI:** 10.1186/s13018-020-01763-x

**Published:** 2020-07-03

**Authors:** Hanru Ren, Rongguang Ao, Lianghao Wu, Zheng Jian, Xinhua Jiang, Baoqing Yu

**Affiliations:** Department of Orthopaedics, Shanghai Pudong Hospital, Fudan University, Pudong Medical Center, No. 2800, Gongwei Road, Shanghai, 201399 China

**Keywords:** Lesser trochanter fracture, Morphologic Analysis, Telescoping, Neck-shaft angle, CT 3D reconstruction

## Abstract

**Background:**

This study investigated the effects of posteromedial fracture fragments on the postoperative stability of intertrochanteric fractures of the femur by analyzing the quantity and range of fragments in CT 3D reconstruction.

**Materials and methods:**

Patients diagnosed with femoral lesser trochanter fractures were collected from September 2015 to February 2018. CT 3D reconstruction was applied to evaluate the quantity and extension of posteromedial fragments and the presence of isolated medial fragments. The stability of postoperative fracture was evaluated by comparing the changes of “neck-shaft angle” and “telescoping” from 1 week to 1 year after operation.

**Results:**

A total of 143 patients were finally confirmed, in which 63 patients contained isolated fragments on the medial side, and the average number of fragments in the posteromedial side was 1.93 ± 0.34, which accounted for an average of about 86.11% ± 8.20% in the whole posteromedial wall. When the number of posteromedial fragments was > 2 and the range of posteromedial fragments was > 75%, then the changes in the neck-shaft angle and “telescoping” showed statistical significance (12.27 ± 4.18 mm and 10.13 ± 6.17°, respectively), and when there were isolated medial isolated fragments, then the change in the neck-shaft angle was 10.66 ± 4.27°, showing statistical significance.

**Conclusions:**

These findings revealed a certain correlation between the quantity and the range of posteromedial fragments and the postoperative “shortening” and “collapse” of femoral intertrochanteric fractures.

## Introduction

Femoral intertrochanteric fracture is the most common type of osteoporotic fracture of the hip, and it most often occurs in elderly (average age 70 years) females [1, 2]. Such patients are confined to bed, and due to no off-bed activity, severe complications are developed, and a high rate of mortality is experienced in them [3]. In short, it is the responsibility of the orthopedician to maintain off-bed activities at an early stage in such patients in order to recover the hip joint function [4].

The lesser trochanter of the femur is at the posteromedial side of the proximal end of the femur, and fracture of this bone often includes comminution of the bone at the posteromedial side [5]. X-ray examination is one of the most frequently used means for detecting femoral intertrochanteric fractures but also forms an important basis in fracture classification [6, 7]. However, with the popularization of CT and 3D reconstruction techniques and new techniques in computer science into clinical results continuously, X-ray examination has been reported to be associated with increasing limitations [8–12]. Although the lesser trochanteric fractures can be observed preoperatively, the size and the comminution degree of the posteromedial bone fragments are poorly assessed, and so it is impossible to completely understand the fracture condition and make surgical planning [13, 14]. In addition, the most common treatment for intertrochanteric fracture is a closed reduction and intramedullary nailing. However, the lesser trochanter of the femur is located in the posteromedial part of the proximal femur, and reduction of it with intramedullary nailing is relatively difficult. Therefore, the isolated lesser trochanter fragment is often not fixed during operation [15]. However, the lesser trochanteric fractures often suggest defects at both medial and posterior cortices, and the intertrochanteric stability is mostly influenced by the medial and posterior cortices. Some studies have suggested that intramedullary nails should be used with extreme caution during the treatment of such fractures, as at times, these require open reduction to ensure better postoperative function.

In this article, we will focus on the lesser trochanteric fractures and the influence of posteromedial fragments. For example, Ehrnthaller et al. have believed that refixation of the lesser trochanter might increase the primary stability of an intertrochanteric fracture [16]. However, there are relatively fewer studies on this research topic, and no studies have further analyzed the effect of lesser trochanteric fractures and their associated posteromedial fragments on the stability after intramedullary nailing of intertrochanteric fractures.

## Materials and methods

This retrospective study received ethics approval from our institution and analyzed patients diagnosed with senile intertrochanteric fractures and include lesser trochanteric fragments by CT 3D reconstruction from September 2015 to February 2018. The inclusion criteria were as follows: (1) patient’s older than 60 years; (2) patients with lesser trochanteric fragments by CT examination; (3) the time from injury to surgery was < 2 weeks for fresh fractures; and (4) patients with closed fracture. Exclusion criteria were as follows: (1) patients with intertrochanteric fracture of type AO-3, in which the fracture line passes through the lateral cortex; (2) intertrochanteric fracture combined with multiple injuries; (3) congenital hip dysplasia; (4) severe osteoporosis; (5) severe medical illness before injury that make them more difficult to walk (tumor, Parkinson); and (6) follow-up time of less than 1 year.

All patients were first diagnosed with femoral trochanteric fracture by emergency X-ray, and then 3D CT reconstruction was carried out for a definitive diagnosis to determine the type of the fracture and judge its range. Surgery within 5 days of admission should be conducted in these patients based on the proximal femoral nail antirotation (PFNA) intramedullary nail closed reduction and internal fixation without fixation of the fracture fragments. Standard rehabilitation exercise was implemented after the operation, i.e., instead of simply lying in bed, patients were given functional exercises, without load walking, and loaded walking exercises were gradually begun after 1 month.

### Fracture reconstruction and calculation of fragment area

CT data were exported in the format of dcm and used in fracture reconstruction. The displacement fracture fragments need to be reconstructed separately and then counted. Moreover, the reconstructed models can be imported into 3-matic software in the format of stl to get the range of posteromedial fragments by calculating the area of posteromedial fragments and the total posteromedial area(Mimics 21.0; 3-matic 13.0).

**Fig. 1:**
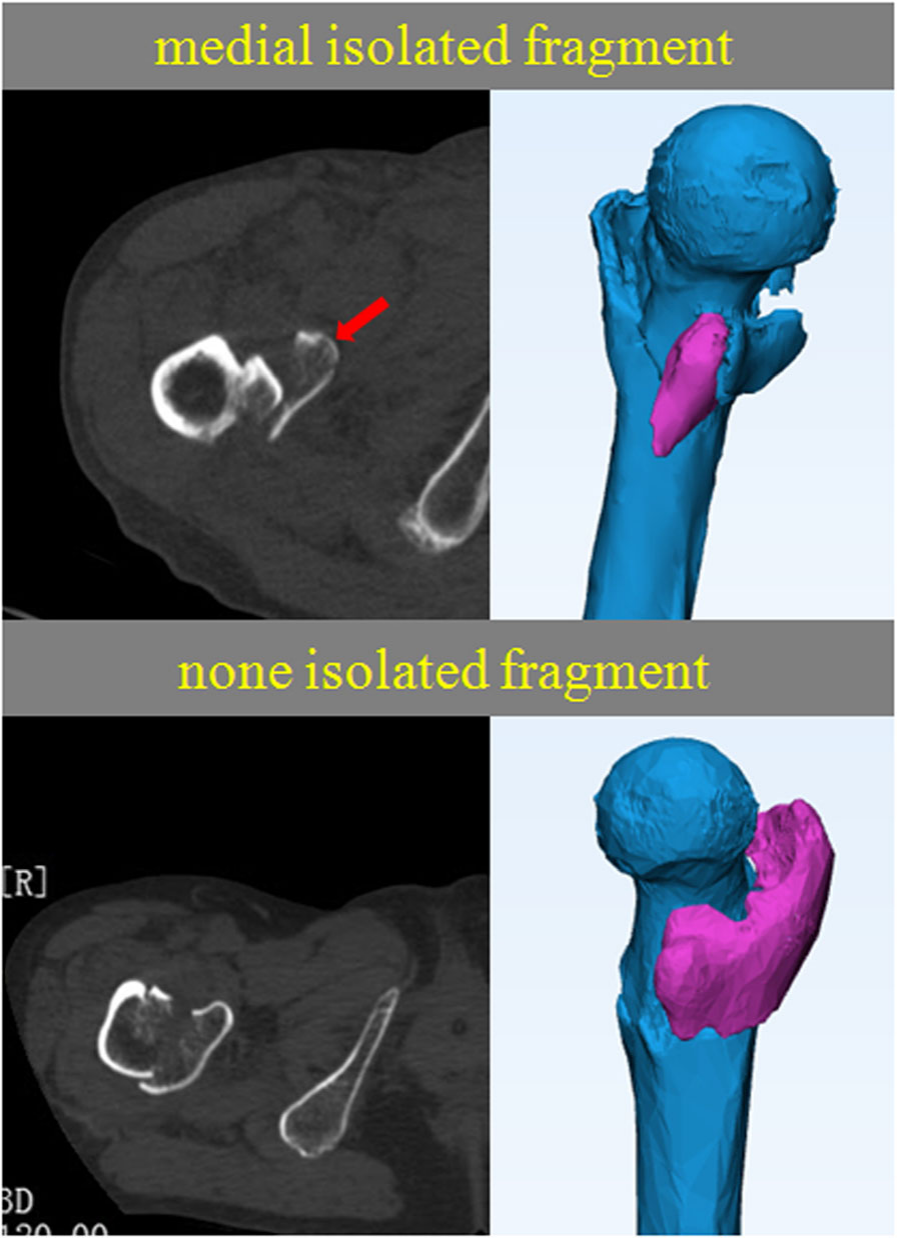
The red arrow shows medial isolated fragment

**Fig. 2:**
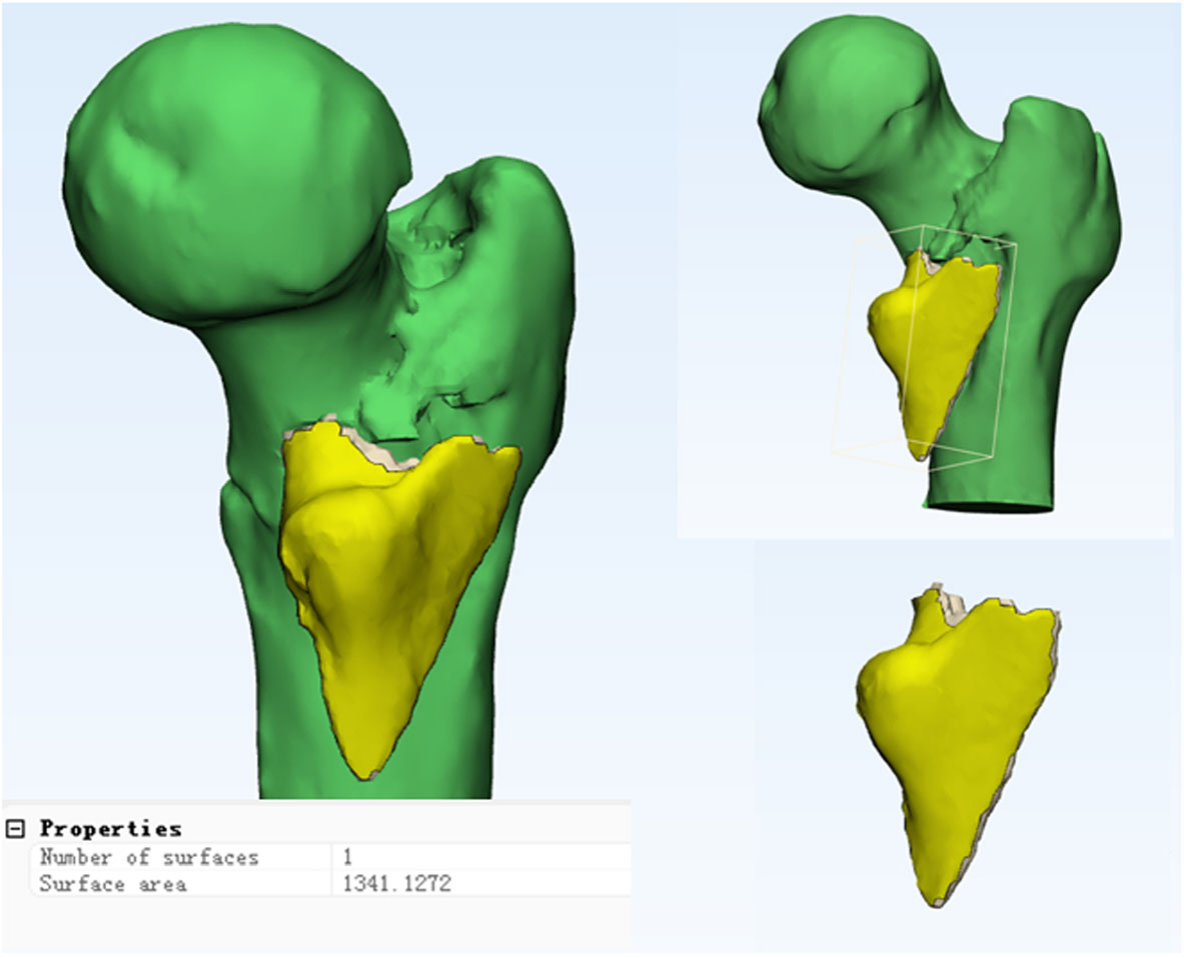
The assessment of quantity and range of patient’s posteromedial fragments. The range was measured by the 3-matic Medical 13.0 software to calculate the femur intertrochanteric total area of the posteromedial and the area of the fragment, respectively; area percentage = area of the posteromedial fragment/total area of the posteromedial. Yellow is the posteromedial lesser trochanter isolated fragment with a surface area of 1341 mm^2^. The posteromedial area manifests only 1 fragment

The isolated medial fragment (Fig. 1) and the quantity and range of posteromedial fragments (Fig. 2) in all the patients were judged by two senior attending physicians from traumatology department by 3D CT reconstruction, and the results have been shown.

### Stability

**Fig. 3:**
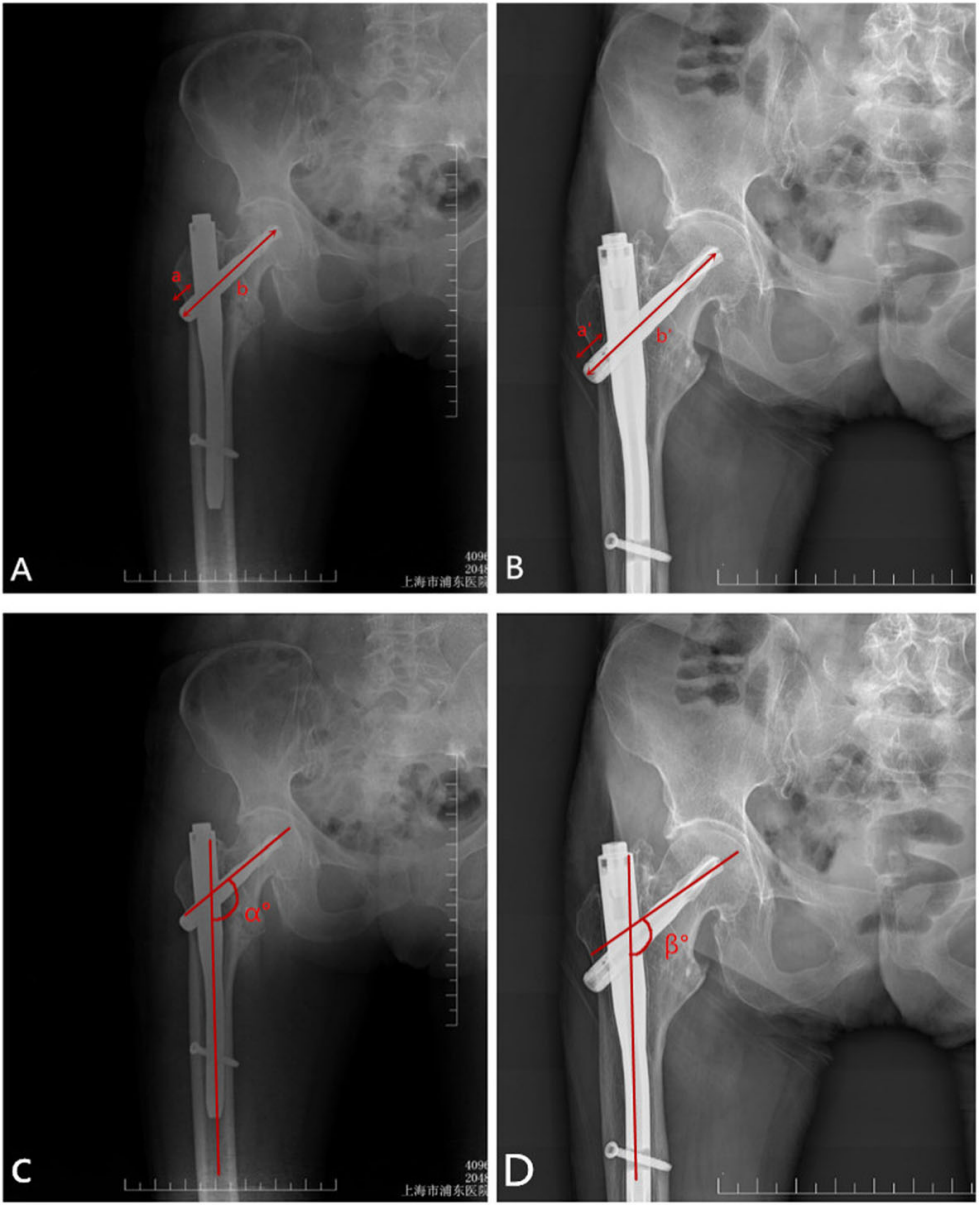
Measurement of telescoping and femoral neck shaft angle changes. Immediate postoperative AP view (**a** and **c**) and last follow-up (postoperative 12 months) AP view (**b** and **d**). Telescoping = measured telescoping (a′) × true blade length/measured blade length (b′) − measured telescoping (a) × true blade length/measured blade length (b). The femoral neck shaft angle change = α° − β°. Measurement of the femoral NSA from DXA scan printouts

Stability after femoral intertrochanteric fracture was judged by “telescoping” and a change in the neck-shaft angle [17, 18]. “Telescoping” quantifies the degree of femoral neck shortening after fracture, and its size is determined by measuring the displacement of the screw blade of the PFNA relative to the lateral cortex in the anteroposterior view. The follow-up time of the patients in this study was 1 week and 12 months after surgery. The displacement degree 1 and displacement degree 2 of the spiral blade of the patient relative to the lateral part of the main nail were evaluated by measuring the standard anteroposterior position of the patient. The displacement degree 2 minus the displacement degree 1 was determined by “telescoping.” If the value of “telescoping” is larger, then its stability of the patient is considered to be poor. The neck-shaft angle can be used to assess the degree of collapse of the femoral head after fracture. The change in each patient’s neck-shaft angle 1 week and 12 months after surgery was determined by applying Hologic 1000 DXA bone densitometry analysis, which is the international standardized measurement (Fig. 3). In addition, the tip-apex distance (TED) in the patient 1 week after surgery was also measured, as it indicates the stability of repair in the intertrochanteric fractures.

All statistical analyses were performed using the SPSS 22.0 statistical software (SSPS, Chicago, IL). The measurement data are presented as means and standard deviations. Changes in the neck-shaft angle and telescoping were analyzed using independent sample *t* test. A *P* value of less than 0.05 was considered to be statistically significant for all tests.

## Results

A total of 256 patients with femoral intertrochanteric fractures were included, and 192 of these were diagnosed as senile patients with lesser trochanteric fragments by CT examination. Of these, 7 patients had multiple other injuries; 3 patients lost muscle force in the lower limbs due to cerebral infarction prior to injury; 21 patients were confirmed dead due to complications within 1 year of surgery; the postoperative TAD was > 25 mm in 6 patients; and 21 patients were lost to follow-up, leaving 143 patients finally. The reasons for injury included falling down at home (102 patients) and traffic accidents (41 patients). Their age, gender, weight, admission time, TAD, and follow-up time are shown in Table 1. The average age of these patients was 73.5 ± 5.12 years, and the follow-up time was 16.12 ± 3.17 months.

**Table 1:** Demographic data and baseline characteristics

	Case (*n*)
Age (years)	73.5 ± 5.12
Gender (male/female)	62/81
Weight (kg)	65.11 ± 7.21
Length of stay (day)	7.52 ± 2.20
Follow-up (month)	16.12 ± 3.17
TAD (mm)	17.23 ± 2.54

In total, 143 patients with femoral intertrochanteric fractures were included, in which 48 patients had 1 posteromedial fragment, 52 with 2 posteromedial fragments, and 43 with fragments larger than 2. In addition, there were 63 patients with isolated medial fragment. The extent of posteromedial fragment was generally large, accounting for approximately 86% of the entire posteromedial wall, which was greater than 75% in 81 patients and 50% to 75% in 27 patients (Table 2).

**Table 2:** The characteristics of LT fragment for patients

Variable	Measurement
Losteromedial fragment range	76.11% ± 8.20%
Posteromedial fragment range < 50%	35
Posteromedial fragment range 50–75%	27
Posteromedial fragment range ≥ 75%	81
Posteromedial fragment quantity	1.93 ± 0.34
Posteromedial fragment quantity = 1	48
Posteromedial fragment quantity = 2	52
Posteromedial fragment quantity ≥ 2	43
Isolated medial fragment	63/143

**Fig. 4:**
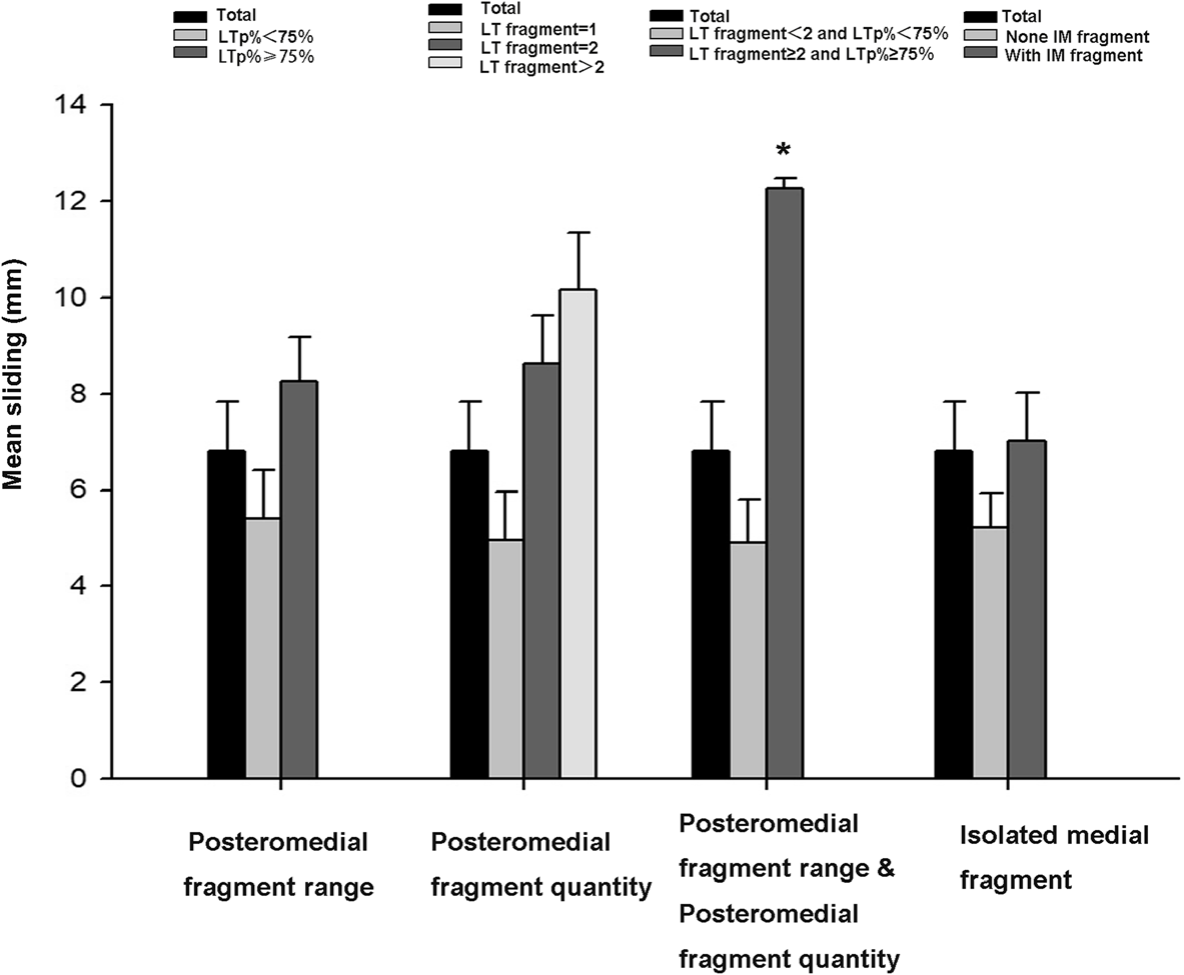
Changes in patient’s “telescoping” at 1 year postoperatively at different posteromedial fragment quantities, range, and presence of separate isolated fragments. When the posteromedial fragments are greater than two and the posteromedial fragments range from was greater than 75%, then the average telescope was 12.27 ± 4.18 mm, with a *P* value of 0.01

The average telescoping of the lateral cortex lag screw at the proximal end of the femur was 6.82 ± 3.21 mm. When the posteromedial fragments are ≤ 2, then the average telescoping was 4.97 ± 3.61 mm and 8.62 ± 3.32 mm; when the posteromedial fragments are greater than two, then the average telescoping was 10.14 ± 5.11 mm, and the difference showed no statistical significance (0.07). When the posteromedial fragment range was ≤ 75%, then the average telescoping was 5.41 ± 2.79 mm, and when the posteromedial fragment range was > 75%, then the average telescoping was 8.27 ± 3.13 mm, which showed no statistical difference (*P* > 0.37). However, when the posteromedial fragments are > 2 and the posteromedial fragment range was > 75%, then the average telescoping was 12.27 ± 4.18 mm, showing statistical difference (*P* < 0.01, Table 3). In addition, we also found that the mean telescopic distance was 7.92 ± 4.18 mm in the presence of an isolated medial fragment, showing no statistical difference (*P* > 0.15, Table 4, Fig. 4).

**Table 3:** Result of the change in neck shaft angle and telescoping for patients between 1 week and 12 months

	Mean sliding (mm)		Neck length ratio (°)	
Total	6.82 ± 3.21		7.22 ± 3.91	
	**Posteromedial fragment range**			
	‾X ± *S*	*P*	‾X ± *S*	*P*
LTp% < 75%	5.41 ± 2.79	0.46	6.92 ± 2.53	0.49
LTp% ≥ 75%	8.27 ± 3.13	0.37	8.13 ± 4.16	0.31
	**Posteromedial fragment quantity**			
	‾X ± *S*	*P*	‾X ± *S*	*P*
LT fragment = 1	4.97 ± 3.61	0.11	5.16 ± 2.13	0.25
LT fragment = 2	8.62 ± 3.32	0.58	7.11 ± 3.21	0.61
LT fragment > 2	10.14 ± 5.11	0.07	9.04 ± 6.37	0.23
	**Posteromedial fragment range and posteromedial fragment quantity**			
	‾X ± *S*	*P*	‾X ± *S*	*P*
LT fragment < 2 and LTp% < 75%	4.91 ± 2.88	0.09	4.92 ± 2.21	0.11
LT fragment ≥ 2 and LTp% ≥ 75%	12.27 ± 4.18	0.01*	10.13 ± 6.17	0.02*

*LT* lesser trochanter

*P < 0.05 was considered significant

**Table 4:** Result of the change in neck shaft angle and telescoping for patients between 1 week and 12 months

	Mean sliding (mm)		Neck length ratio (°)	
Total	6.82 ± 3.21		7.22 ± 3.91	
	**Isolated medial fragment**			
	‾X ± *S*	*P*	‾X ± *S*	*P*
None IM fragment	5.23 ± 0.88	0.46	5.57 ± 2.43	0.30
With IM fragment	7.92 ± 4.13	0.15	10.66 ± 4.27	0.01*

*IM* isolated medial

*P < 0.05 was considered significant

**Fig. 5:**
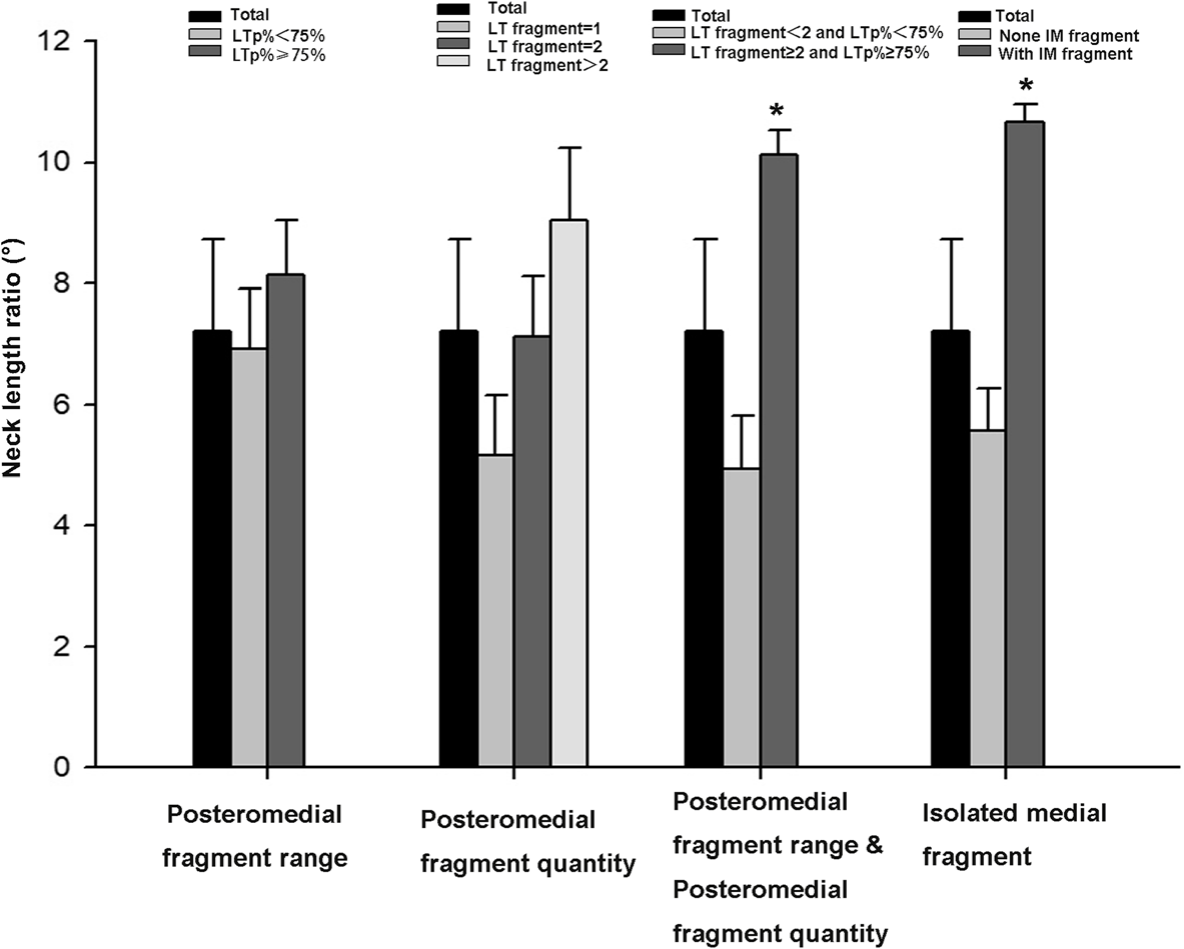
Changes in patient’s “neck-shaft angle” at 1 year postoperatively with varying amounts, extent, and presence of isolated fragments in the posteromedial aspect. *P* value was less than 0.05 when the posteromedial fragments are greater than two, and the posteromedial fragments range was greater than 75% or when the posteromedial independent fragment exists

The average change in the neck-shaft angle was 7.22 ± 3.91°. When there are only one or two posteromedial fragments, the changes in the neck-shaft angle are 5.16 ± 2.13° and 7.11 ± 3.21°, and when there are more than two fragments, the average change in the neck-shaft angle was 9.04 ± 6.37°, with no statistical differences (*P* > 0.23). When the posteromedial fragment range was ≤ 75%, then the average change in the neck-shaft angle was 6.92 ± 2.53°, and when the posteromedial fragment range was > 75%, then the average change in the neck-shaft angle was 8.13 ± 4.16°, with no significant difference (*P* > 0.31). However, when the posteromedial fragments are > 2 and the posteromedial fragment range was > 50%, then the change in the neck-shaft angle was 10.13 ± 6.17°, with a statistically significant difference (*P* < 0.025, Table 3). However, the mean neck-shaft angle change was 10.66 ± 4.27 in the presence of an isolated medial fragment, showing statistically significant differences (*P* < 0.01, Table 4, Fig. 5).

## Discussion

Intertrochanteric fractures of the femur are still considered as “unresolved fracture” types due to their association with high postoperative complications and mortality [4]. According to the biomechanical research, the lateral wall of the femoral trochanter is the tension side, while the medial wall is the pressure side, and so it easily causes femoral head introversion and shortens the deformity of femoral neck in case of medial and posteromedial comminuted fracture with fragments, causing extremely unstable fractures [19]. Of course, we also agree that the software tools do not replace experimental testing, but they provide a valuable and rapidly evolving option [20]. Intramedullary, nailing is a common surgical method for treating intertrochanteric fractures, but according to the study by Parker et al., intramedullary nailing for unstable intertrochanteric fractures more likely leads to adverse complications. However, the posteromedial fragment is rarely fixed during the surgery so far, and the reduction of neck-shaft angle after surgery still remains very common and is one of the important reasons for the functional impact of intertrochanteric fractures after surgery [21].

First of all, the TAD was measured in all patients by X-ray at 1 week after surgery, and this should take into consideration that different operators and differences in the quality of surgical reduction might lead to postoperative “telescoping” and changes in neck-shaft angle. When TAD was < 25 mm, then the insertion angle and depth of screw blade of intramedullary nail are relatively satisfactory, which are within the acceptable range and included in our study.

In addition, the effect of postoperative stability of femoral intertrochanteric fracture was assessed by evaluating the changes of neck-shaft angle and telescoping. This is because we believed that femoral head collapse and femoral neck shortening are the common causes of postoperative reduction failure of femoral intertrochanteric fractures.

Due to the two causes, the stability of the patient after surgery might be affected, and femoral head collapse more likely leads to a cut out. Cut out refers to the phenomenon where the screw blade is displaced upwards and the femoral head is cut out after the fracture surgery, while femoral neck shortening is related to excessive lateral slip of screw blade, which is referred to as the “telescope effect”. Severe telescope effect also leads to internal fixation failure [22]. We believed that the process of femoral head collapse must be associated with postoperative changes in the size of the neck-shaft angle. If the quality of fracture reduction is insufficient and the stability is lacking, the neck-shaft angle is further reduced and causes continuous collapse of the femoral head, eventually leading to the occurrence of cut out in patients. Neck shortening can be assessed by measuring “telescoping” change. If the postoperative stability is poor, then the femoral neck was continuously shortened as the screw blade continuously slides laterally. Therefore, we believed that the postoperative stability of intertrochanteric fracture can be more effectively evaluated by combining the neck shaft angle and telescoping changes.

It is well known that the integrity of the posteromedial wall of the femoral intertrochanter plays an important biomechanical role to ensure the stability of the proximal femur [23]. The loss of posteromedial support is considered to be an important cause of femoral head collapse, femoral neck shortening, internal fixation failure, and even secondary surgery. Some scholars have proposed that the concept of positive support during intramedullary nail treatment prevents the reduction of postoperative neck-shaft angle [24]. However, it is difficult to obtain a satisfactory “positive support” during operation due to comminuted fracture on the posteromedial wall and the surgical method of closed reduction.

We consider that the quantity and range of posteromedial fragments might be equally important in the stability of the medial wall of the intertrochanter. Although our study showed no significant correlation between the quantity of single fragment and the range of fragment and “shortening” and “collapse” of the femoral head (this result was similar to some of the previous results), the change in the neck-shaft angle and the change in the telescoping of postoperative patients were significantly smaller when the medial fragments were smaller and the range of fracture was smaller, which might be due to insufficient sample size. Also, the incidence of “shortening” and “collapse” after intertrochanteric fracture surgery was significantly higher when the quantity of fragments was greater than 2 and the range of fragments was greater than 50%. We believed that this might be associated with several reasons: (1) when there are more quantities and more range of posteromedial fragments, then it effects on the quality of reduction during surgery, the occurrence of postoperative isolated fragments, and loss of support on the medial side; (2) although there are several studies showing that the isolated fragments at the femoral intertrochanteric gradually reduces itself with contraction force of the muscle, the time of reduction often takes more than 8 months. As the medial wall plays a relatively important supporting role, it might lead to the collapse of the femoral head and even cut out during the process of walking and gradually increasing the weight bearing exercises after 1 month; (3) in addition to major violence, the comminuted posteromedial fracture might be closely related to the osteoporosis of the patient, which further aggravates osteoporosis in the process of fracture surgery and rehabilitation, while we believed that there is a correlation between severe osteoporosis and the occurrence of “shortening” and “collapse” after intertrochanteric fracture.

After trochanteric fractures, the presence of isolated fragments on the medial side affects the stability? The results of our study demonstrated a significant correlation between the change in independent medial fragments and neck-shaft angle but not with the change in telescoping. The reason as to why the independent medial fragment as an observation indicator was initially used in this experiment was that when only intramedullary nailing was used for fixation during traditional surgical method, then the phenomenon of isolated fragment often occurred in the medial independent fragment. Also, there was also a defect in the medial cortex accompanied by the isolated fragment, and this might be closely related to the postoperative femoral head collapse. Therefore, there was a significant correlation between the indicator of change of neck-shaft angle and the independent medial fragment, but there was no correlation between femoral neck shortening corresponding to the telescoping change and the occurrence of independent medial fragment. So, the close relationship between the occurrence of femoral neck shortening and the intraoperative and postoperative risk factors requires further exploration.

## Conclusion

This article investigated the effect of posteromedial fragments on the postoperative stability of intertrochanteric fractures. The results showed that the quantity of posteromedial fragments and the range of fragments showed correlation with postoperative “shortening” and “collapse” of intertrochanteric fractures. In addition, when the quantity of posteromedial fragments was more than 2 and the range of fragments was greater than 50%, then the incidence of postoperative “shortening” and “collapse” of intertrochanteric fractures was significantly higher, and the stability was poor. However, the presence of medial independent fragments was significantly correlated with the postoperative neck-shaft angle changes and femoral head collapse.

In future work, whether the internal fixation such as auxiliary steel cable can be used in patients with CT confirmed posteromedial comminuted fractures or whether it is necessary to delay the time of weight-bearing training after operation to ultimately reduce the incidence rate of severe complications of such fractures still require exploration.

## Data Availability

The datasets used and analyzed during the current study are available from the corresponding author on reasonable request.

## Abbreviations

3D: Three-dimensional
CT: Computed tomography
PFNA: Proximal Femoral Nail Antirotation
TAD: Tip-apex distance
LT: Lesser trochanter
